# The Task Force for Child Survival: Secrets of Successful Coalitions

**DOI:** 10.3201/eid2504.181819

**Published:** 2019-04

**Authors:** Jane M. Gould

**Affiliations:** Pennsylvania Department of Health, Harrisburg, Pennsylvania, USA

**Keywords:** book review, task force, vaccine programs, global health, child survival, vaccination, coalitions

Well-written and inspirational, *The Task Force for Child Survival: Secrets of Successful Coalitions* (Figure) is a firsthand perspective on the creation and history of one of the most successful coalitions in global health. An expert authority on vaccine programs and global health, author William H. Foege, former director of the US Centers for Disease Control and Prevention, was the lead architect of the Task Force. Formed in 1984, the goal of the Task Force was to facilitate the work of the World Health Organization and UNICEF to improve global immunization coverage in children. At that time, 4.3 million deaths occurred per year, >12,000 children per day, from vaccine-preventable diseases measles, tetanus, and pertussis alone. To increase vaccine coverage, the Task Force had to overcome several complicated logistical issues, have an optimistic outlook despite the reality of what was feasible, and have the dedication of passionate individuals working as a team. Increasing vaccination coverage was a daunting task, yet they achieved success where other organizations had failed. 

**Figure:**
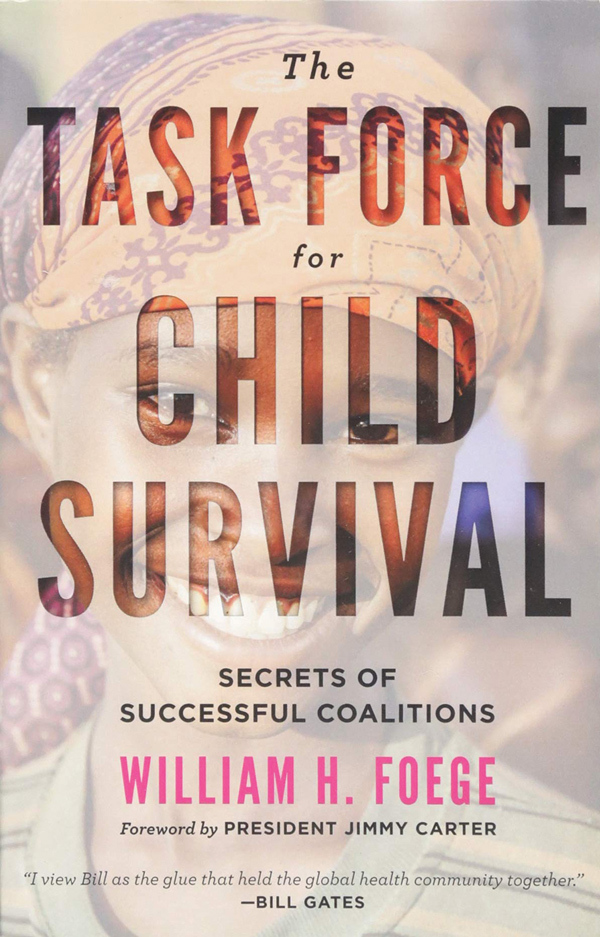
The Task Force for Child Survival: Secrets of Successful Coalitions

The Task Force also worked to increase access to Mectizan, a preventive medicine for onchocerciasis, also known as river blindness. Previous programs sought to control the black flies that transmit the disease-causing parasitic worm *Onchocerca volvulus*, without much success. The historical description of the Mectizan program, including how the drug was developed and donated free of charge by Merck until the disease is eliminated, is captivating reading. The Task Force worked alongside Merck and became a crucial partner in the distribution and oversight of the drug, including surveillance for side effects. The program grew as the drug was shown to be safe and effective, reaching 6 million people within 4 years, and eventually 20 million people per year, with assistance from the World Bank.

A chronological account of the Task Force is the first aim of the book, which will be fascinating reading for those training or already working in global health, public health, infectious diseases, or immunization programs. The author’s second purpose is to describe how successful coalitions develop and function. His insights are instructive reading for anyone currently a member of or tasked with creating a coalition or a collaborative endeavor. The short chapter “How Productive Coalitions Begin” and another, “Lessons Learned,” contain crucial messages for building and sustaining functional coalitions, such as obtain commitment of partner agency leaders; have a shared, well-defined, and attainable goal (in this case 80% of the world’s children would receive at least 1 vaccine by 1990); get input from those in the trenches on major barriers; prioritize good communications with all stakeholders; and emphasize the need for “ego suppression.” Dr. Foege stresses that “guarding turf, seeking credit or becoming the spokesperson is hazardous to the health of the coalition.” His description of great leadership and how successful coalitions and collaborations function underscores how vital both are to solving future problems in public health.

